# Enhanced neuromorphogenesis of neural stem cells via the optimization of physical stimulus-responsive signaling pathways

**DOI:** 10.1186/s13287-025-04488-y

**Published:** 2025-07-18

**Authors:** Youyi Tai, Natasha Brinkley, Lu Jin, Yu Wei Chang, Allen Liakhovetski, Jin Nam

**Affiliations:** Department of Bioengineering, University of California, Riverside, CA USA

**Keywords:** Neural stem cell differentiation, Mechano-electrical stimulation, Neuromorphic signaling pathway, Nerve tissue model

## Abstract

**Background:**

Neural stem cells hold significant promise for developing in vitro nerve models due to their capacity to differentiate into diverse neural cell types. While traditional biochemical approaches often restrict differentiation to a single phenotype, limiting the ability to study critical neuron-glia interactions, physical stimuli have been explored due to their capacity to drive multi-phenotypic differentiation of neural stem cells. However, underlying molecular mechanisms mediating the physical stimulation-induced neural stem cell differentiation, with an emphasis on electrical stimulation and mechanical stimulation, remain inadequately explored, hindering the comprehensive optimization and application of physical stimulation for enhanced neuromorphogenesis.

**Methods:**

In this study, we explored the signaling pathways driving mechano-electrical stimulation-induced multi-phenotypic differentiation of NSCs using a piezoelectric platform with signaling inhibitors. The signaling knowledge was then used to further enhance the neuromorphogenesis by employing signal activator/inhibitor in combination with mechano-electrical stimulation.

**Results:**

We discovered that electrical stimulation promotes the neuronal differentiation of NSCs via Wnt signaling through the TRPC1 channel, while mechanical stimulation activates the TRPV4-RhoA/ROCK axis, inducing astrocytic and oligodendrocytic differentiation via JAK/Stat3 and Shh/Gli1 pathways, respectively. Targeted modulation of these pathways under mechano-electrical stimulation further enhanced neuromorphogenesis, including improved neurite outgrowth, synaptic interactions, and myelin maturation.

**Conclusions:**

This study systematically uncovered the signaling cascades that mediate the mechano-electrical stimulation-induced multi-phenotypic differentiation of neural stem cells towards neurons and glial cells, which were separately mediated by electrical stimulation and mechanical stimulation, respectively, through independent signaling cascades. The combination of physical stimulation with biochemical factors to modulate those signaling pathways further enhanced neuromorphogenesis, offering a reliable and robust strategy to develop fully functional neural stem cell-derived in vitro nerve models.

**Supplementary Information:**

The online version contains supplementary material available at 10.1186/s13287-025-04488-y.

## Background

During development, neural stem cells (NSCs), originating from the ectoderm, differentiate into diverse functional phenotypes within nerve tissues, including neurons, astrocytes, and oligodendrocytes, contributing to the development of the central nervous system (CNS). In addition to their involvement in CNS development, a small population of NSCs persists in mature tissues, playing a critical role in maintaining CNS functionality during disease and/or following injuries [1–3]. Capitalizing on their capacity for diverse phenotypic differentiation and functional nerve tissue formation, NSCs have been utilized to create various in vitro nerve tissue models. These models serve as valuable tools for investigating numerous neurological diseases and syndromes, facilitating drug discovery/screening, and advancing our understanding of functional nerve regeneration following injury [4–6]. Most of these in vitro nerve models employ biochemical mediators to differentiate NSCs toward specific neural cell phenotypes. For example, the removal of epidermal growth factor and basic fibroblast growth factor from normal neural stem cell growth media results in the neuronal differentiation of the cells while the addition of platelet-derived growth factor induces the differentiation of NSCs towards glial cells [7–9]. In addition, insulin-like growth factor accelerates the differentiation of NSCs towards oligodendrocytes [10]. While these biochemical factor-mediated methods for controlling NSC differentiation are well-established, the prevailing approach in developing NSC-derived nerve models often involves utilizing a single-cell type or employing simplistic co-culture systems. Unfortunately, this strategy tends to overlook the importance of addressing physiologically relevant cell–cell communications, specifically neuron-glial interactions [11, 12]. Consequently, these models often fall short of achieving the desired level of robustness, replicability, and predictiveness required for effective in vitro models of nervous microphysiological systems.

In this regard, the application of physical stimuli including electrical and mechanical stimulation has been shown to significantly affect the differentiation/maturation of NSCs to multiple neural cell types [13–16], substantiating the critical role of the physical microenvironments in nerve tissue development/regeneration [17, 18]. For example, electrical stimulation has been shown to induce simultaneous differentiation of NSCs toward mature neurons and astrocytes [13]. Similarly, neuronal and astrocytic differentiation of NSCs was also observed under mechanical strain by stretching a cell-culture substrate [19]. Furthermore, it was recently demonstrated that appropriate mechano-electrical stimulation (MES), generated by acoustic activation of a piezoelectric scaffold, induces the multi-phenotypic differentiation of NSCs towards neurons, astrocytes, and oligodendrocytes, resulting in the formation of self-organized nerve tissue [20]. While these observational insights underscore the impact of physical stimuli on NSC differentiation and the morphogenesis/regeneration of nerve tissue, the underlying molecular mechanisms guiding the differentiation induced by such physical stimuli remain inadequately explored. This knowledge gap currently hampers the comprehensive optimization and application of physical stimulation for enhanced neuromorphogenesis, crucial for engineering in vitro nerve tissue models and its adoption to in vivo nerve regeneration.

To methodically maximize functional nerve tissue morphogenesis from NSCs, our study aimed to (1) identify major signaling networks involved in MES-induced neuro-, astro-, and oligodendrogenesis; (2) utilize such signaling knowledge to further enhance nerve tissue development. Based on prior findings regarding various signaling cascades involved in neuromorphogenesis, including Wnt/β-catenin, Janus kinase (JAK)/Stat3, and Sonic hedgehog (Shh)/Gli1 signaling pathways [21–27], we systematically examined whether these signaling cascades mediate MES-induced neuronal and glial differentiation of NSCs. Furthermore, we unveiled the upstream physico-responsive transient receptor potential (TRP) channels involved in the MES-induced differentiation of NSCs. Motivated by these findings, selective activation/deactivation of those signaling cascades was utilized to further improve the morphogenesis and functionality of an NSC-based in vitro nerve tissue model, resulting in significantly enhanced neurite length, enriched synaptic network formation, and increased mature neuronal-glial interface. This systematic study of physical stimuli-mediated NSC differentiation/maturation, therefore, provides a means to optimize in vitro neuromorphogenesis and potentially offers a reliable platform for developing nerve disease/injury models.

## Methods

### Synthesis of poly(vinylidene fluoride-trifluoroethylene) (P(VDF-TrFE)) and polyvinylidene fluoride (PVDF) scaffolds

The electrospinning technique was used to fabricate piezoelectric P(VDF-TrFE) scaffolds as described previously [20]. Briefly, 7 wt% of P(VDF-TrFE) (70:30 mol%, PolyK) was dissolved in a solvent system containing a 60/40 volume ratio of N,N-dimethylformamide (Sigma) to methyl ethyl ketone (Sigma), supplemented with 1 wt% pyridinium formate buffer (Sigma). Alternatively, a 13.5 wt% solution of PVDF (Sigma) dissolved in the same solvent system was utilized for non-piezoelectric control samples. The solutions were electrospun to produce a fiber diameter of 523 ± 77.45 nm with the optimized spinning conditions, including 10 cm of tip-to-collector distance, 15 kV applied voltage, 7.6 g m^−3^ of absolute humidity, and 6 ml h^−1^ of solution feed rate. Electrospun fibers were collected onto a rotating wheel at an angular velocity of 47.9 m s^−1^ to yield an aligned fibrous structure and the spinning duration was optimized to produce 202 ± 20 µm thickness. For the non-piezoelectric controls, electrospun PVDF fibers were thermally treated at 157 °C for 1 h in a rapid thermal annealing oven (Allwin21 Corp) to deactivate piezoelectricity by facilitating the transition from the electroactive phase to the non-piezoelectric α-phase without melting the fibrous structure. This was immediately followed by quenching in cold ethanol to stabilize the non-piezoelectric α-phase. The morphology and piezoelectric properties of the electrospun fibers were characterized as described elsewhere [20].

### Cell culture of mouse and human neural stem cells

C17.2 mouse neural stem cells (mNSCs), derived from the cerebellum of a neonatal mouse [28], were cultured in high glucose DMEM (Corning), supplemented with 10% FBS (VWR), 5% horse serum (Gibco), 1% sodium pyruvate, and 1% penicillin/streptomycin (Fisher).

All experiments involving human stem cells were approved by the UC Riverside Institutional Review Board (IRB; HS11-124) and Stem Cell Research Oversight Committee (SCRO; SC20210002). Human neural stem cells (hNSCs) were derived from well-characterized human induced pluripotent stem cells (iPSCs) using a neural induction medium (Gibco) as described previously [20]. The iPSC-derived hNSCs were cultured in an NSC expansion medium containing 50% Neurobasal medium (Gibco), 50% Advanced DMEM/F12 (Gibco), and a Neural induction supplement (Gibco).

### The application of MES

MES was applied using piezoelectric P(VDF-TrFE) scaffolds activated by the hydroacoustic actuation culture system as described previously [20, 29]. Briefly, P(VDF-TrFE) scaffolds were secured in a 3D-printed hollow culture chamber. The scaffold-culture chamber in a 6-well culture plate was placed on a vertical actuation system. A periodic pulse of energy flux (1.226 × 10^−4^ mJ mm^−2^, 3 Hz) was applied through the actuation system to induce an estimate 0.03% strain change on P(VDF-TrFE) scaffolds, resulting in 200 mV_p-p_ voltage output (figure S1A). In this system, mechanical stimulation directly from hydroacoustic actuation and electrical stimulation generated by the actuation of piezoelectric P(VDF-TrFE) would simultaneously affect the cells cultured on the scaffold surface. The cells were allowed to attach and proliferate on P(VDF-TrFE) scaffolds for 48 h before being subjected to MES. The voltage outputs generated from the scaffolds were controlled to be approximately 200 mV_p-p_ under a physiologically safe mechanical stimulation as demonstrated elsewhere [20, 29]. For the demonstration of multi-phenotypic differentiation of NSCs under MES, either mNSCs or hNSCs were daily stimulated for 2 h for 2 weeks, followed by fixation in 4% paraformaldehyde (PFA) for imaging or lysed for gene expression analysis. For the signaling mechanistic study, the mNSCs were either subjected to MES for various durations including 0, 15 min, 30 min, 1 h, or 2 h before fixation in 4% PFA, or lysed at multiple time points including 1, 2, 4, 6, 10 h after the initiation of the 2-h MES. Various signaling activators or inhibitors were applied including β-catenin inhibitor (XAV939, 500 nM, Tocris), Stat3 inhibitor (Cucurbitacin I, JSI124, 0.5 µM, Tocris), Gli1 inhibitor (As_2_O_3_, 4 µM, Sigma), Akt inhibitor (GSK690693, 13 nM, Tocris), ROCK inhibitor (RI, Y-27632, 10 µM, Sigma), TRPC1 inhibitor (Pico145, 2 nM, Tocris), TRPV4 inhibitor (RN 1734, 11.8 µM, Tocris), and TRPM7 inhibitor (Ver 155,008, 10 µM, Tocris). These activators and inhibitors were individually applied either during MES (inhibitors) or under normal culture conditions (activators). To demonstrate the enhancement of neuromorphogenesis under the combination of biomechanical and biochemical stimulation, hNSCs were subjected to MES for an overall duration of 5 weeks. NGF and Y-27632 (ROCK inhibitor) were applied to the system for the first 2 weeks of culture with optimal concentration and duration, which were determined by the Taguchi-based robust parameter design (RPD) analysis.

### The application of electrical stimulation (ES) and mechanical stimulation (MS)

The cellular effects of ES and MS generated by MES through hydroacoustic actuation of the piezoelectric scaffold were decoupled. To isolate the effects of ES, the cell/scaffold construct was placed on a conductive surface (gold-coated polystyrene) within a well of the 12-well tissue culture plate [20, 29]. The bottom of each well was drilled to electrically wire the cell/scaffold construct to a power supply controlled by a function generator. A grounded gold-coated glass coverslip was placed above the gold-coated polystyrene in contact with the cell culture media within each well. An electrical pulse of 200 mV_p-p_ was applied to the cells to match the voltage output of the P(VDF-TrFE) scaffolds under the MES condition (figure S1B). heat-inactivated PVDF scaffolds, which exhibit little or no piezoelectric effect, were used within the hydroacoustic actuation culture system, similar to the MES condition. The piezoelectric inactivation of PVDF was achieved by proper thermal treatment using a temperature slightly above PVDF’s curie point but below its melting point [20]. Such thermal treatment induced a phase transition of PVDF from a piezoelectric active beta-phase to piezoelectric inactive alpha-phase while not significantly affecting the electrospun fibrous structure. As a result, the voltage output of the inactivated PVDF was near 0 under the same amount of applied energy flux (1.226 × 10^−4^ mJ mm^−2^) and strain change (0.03%) (figure S1C). Each stimulus was applied to the cells for 2 h and they were lysed for proneural gene expression using RT-qPCR. Unstimulated cells cultured on P(VDF-TrFE) scaffolds (Static) and cells subjected to MES (MES) were used as control groups.

### Immunofluorescence imaging

PFA-fixed cells were stained with the following primary antibodies anti-Tuj. 1 (Fisher), anti-GFAP (Cell Signaling), anti-GalC (Proteintech), anti-β-catenin (BD biosciences), anti-p-Stat3 (Cell Signaling), anti-Gli1 (Cell Signaling), anti-vGAT (Proteintech), anti-Gephyrin (Santa Cruz Biotechnology), anti-VGLUT1, anti-PSD95 (DSHB), and anti-MOG (Santa Cruz Biotechnology) with appropriate secondary antibodies (Goat Anti-Mouse lgG (H + L) with Alexa Fluro@ 488 or 594 and Goat Anti-Rabbit lgG (H + L) with Alexa Fluro@ 488 or 594, Jackson ImmunoResearch), followed by counterstaining with DAPI (Sigma). A confocal microscope (Zeiss 880) was used to visualize the expression of different markers. ImageJ software was used to quantify the protein expression intensity or protein nuclear localization.

### Gene expression analysis

Total RNA was extracted using an RNeasy Micro Kit (Qiagen) followed by cDNA synthesis using an iScript cDNA Synthesis Kit (Bio-rad). RT-qPCR was performed to determine the gene expression of neurons, astrocytes, and oligodendrocytes phenotypic primer markers (Table S1, Table S2). Raw data were analyzed by the comparative threshold cycle (C_T_) method using the expression of Gapdh as an endogenous control. In addition, PCR electrophoresis was performed to determine the expression of TRP channels in mNSCs using primers listed in Table S3.

### Scanning electron microscopy (SEM)

To examine the morphology of engineered nerve tissues, PFA-fixed cell/scaffold constructs were subjected to a gradient series of ethanol (30%, 50%, 70%, 80%, 95%, and 100%), followed by hexamethyldisilazane (25%, 50%, 75%, and 100%) series. The surface of the dehydrated samples was gold-sputtered (Cressington) and subjected to SEM imaging (name of the instrument).

### Statistical analysis

All experiments were conducted with a minimum of triplicate biological samples and data are presented as mean ± standard deviation or standard error of means, depending on the type of data obtained. Comparison of experimental groups for statistical significance was determined using the IBM SPSS software with either one-way ANOVA with Tukey’s HSD posthoc test or a two-sample student T-test. Statistical significance was reported when a ‘*p*’ value was less than 0.05. Mini-tab statistical analysis software was used for the Taguchi robust parameter design (RPD) analysis. The calculated mean values indicate the effect of each condition on neurite length and myelination percentage. “Larger-the-better” criteria was employed where optimal conditions are determined by selecting levels and factors exhibiting the highest mean value, i.e., the “longest neurite length” in this study.

## Results

### Mechano-electrical stimulation induces multi-phenotypic differentiation of neural stem cells towards neurons, astrocytes, and oligodendrocytes.

**Fig. 1:**
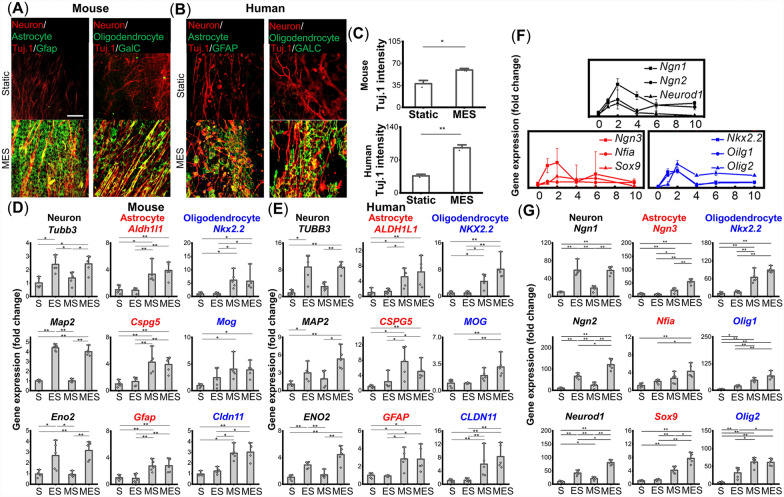
Neural stem cells (NSCs) differentiate simultaneously toward neuronal and glial phenotypes under mechano-electrical stimulation (MES). (**A**, **B**) Immunofluorescence images for the expression of a neuronal marker beta III tubulin (Tuj. 1), an astrocytic marker Gfap/GFAP, and an oligodendrocytic marker GalC/GALC after 2 weeks of static culture or under daily mechano-electrical stimulation (MES; 2 h/day) for (**A**) mouse or (**B**) human NSCs (scale bar: 80 μm). (**C**) Quantification of Tuj. 1 fluorescence intensity for both mouse and human cells (n = 3). (**D**, **E**) Gene expression of (D) mouse or (E) human NSCs for neuronal markers (*Tubb3/TUBB3*, *Map2/MAP2*, *Eno2/ENO2*), astrocytic markers (*Aldh1l1/ALDH1L1*, *Cspg5/CSPG5*, *Gfap/GFAP*), and oligodendrocytic marker (*Nkx2.2/NKX2.2*, *Mog/MOG*, *Cldn11/CLDN11*) after 2 weeks of culture under the Static (S), electrical stimulation (ES), mechanical stimulation (MS), or MES condition (n = 5). (**F**) Time-course study of gene expression for early markers of neurogenesis *Ngn1*, *Ngn2*, *Neurod1*, astrogenesis *Ngn3*, *Nfia*, *Sox9*, and oligodendrogenesis *Nkx2.2*, *Olig1*, *Olig2* at various time points under MES. (**G**) Gene expression levels of the early markers under the S, ES, MS, or MES condition at 2 h after the initiation of MES (n = 5). * and ** denote statistical significance of *p* < 0.05 and *p* < 0.01, respectively

The multi-phenotypic differentiation of NSCs towards neurons and glial cells under the application of physical stimuli is demonstrated in Fig. 1A–E. Both mouse and human NSCs exhibited the expression of a neuronal marker, beta III tubulin (Tuj. 1), either under the Static (cells cultured on P(VDF-TrFE) scaffolds without their piezoelectric activation) or MES (cells cultured on P(VDF-TrFE) scaffolds with daily activation (2 h/day)) condition. The spontaneous neuronal differentiation in the Static condition is likely due to the aligned fibrous morphology of the piezoelectric scaffold, guiding cellular alignment and subsequent neuronal differentiation as previously demonstrated (Fig. 1A, B) [20, 30]. However, MES induced stronger expression of Tuj. 1, based on immunofluorescence quantification, suggesting the role of MES in further enhancing neuronal differentiation (Fig. 1A–C). More importantly, the expression of an astrocyte marker, Gfap/GFAP, and an oligodendrocyte marker, GalC/GALC, was observed only in the cells that were subjected to MES (Fig. 1A, B). In order to further examine the effects of each individual stimulus on neural stem cell differentiation, MES was decoupled into electrical stimulation (ES) and mechanical stimulation (MS) using a non-piezoelectric version of PVDF scaffolds. Gene expression levels of mature neuronal, astrocytic, and oligodendrocytic markers were examined after 2 weeks of culture under various conditions (Fig. 1D, E). The results showed that significant upregulation of neuronal markers (*Eno2*/*ENO2*, *Tubb3*/*TUBB3*, and *Map2*/*MAP2*) was observed under the ES and MES conditions, but not under MS for both mouse and human NSCs. On the contrary, MS and MES significantly enhanced the expression of astrocytic and oligodendrocytic markers (*Gfap/GFAP*, *Aldh1l1/ALDH1L1*, *Cspg5/CSPG5*, *Cldn11/CLDN11*, *Nkx2.2/NKX2.2*, *Mog/MOG*), whereas ES had less impact on the expression of these glial genes (Fig. 1D, E). These results of stimulus type-dependent differentiation led us to hypothesize that ES and MS trigger different signaling cascades to promote the neuronal and glial differentiation of NSCs, respectively.

### Wnt/β-catenin, JAK/Stat3, and Shh/Gli1 signaling pathways mediate electrical and mechanical stimulation-induced neuronal, astrocytic, and oligodendrocytic differentiation, respectively

To further examine the effects of physical stimuli on NSC differentiation comprehensively, the expression of pro-neural transcription factors that initiate neurogenesis and gliogenesis was examined to capture earlier stages of differentiation. To account for the transient nature, the expression levels were examined at multiple time points under MES (Fig. 1F). The maximum upregulation of neuronal transcription factors *Ngn1*, *Ngn2*, *Neurod1*, astrocytic transcription factors *Ngn3*, *Nfia*, *Sox9*, and oligodendrocytic transcription factors *Nkx2.2*, *Olig1*, and *Olig2* coincided with the 2-h time point after the initiation of the stimulation. Based on this observation, the expression level of proneural transcription factors under different stimulation conditions was evaluated 2 h after the initiation of MES for the subsequent gene expression experiments. Consistent with the mature neuronal gene expression data in Fig. 1, we found that the neurogenic transcription factors were significantly upregulated under ES and MES conditions as compared to those under the MS condition (Fig. 1G). Unlike the expression pattern shown in neurogenic transcription factors, the expression levels of astrogenic and oligodendrogenic markers were more significantly upregulated under the MS and MES, supporting our hypothesis that ES enhances neuronal differentiation while MS enhances glial differentiation. It should be noted that the examined transcription factors were the most significantly upregulated under the MES condition as compared to all the other conditions, potentially indicating that there is a synergistic effect between ES and MS on NSC differentiation (Fig. 1G).

**Fig. 2:**
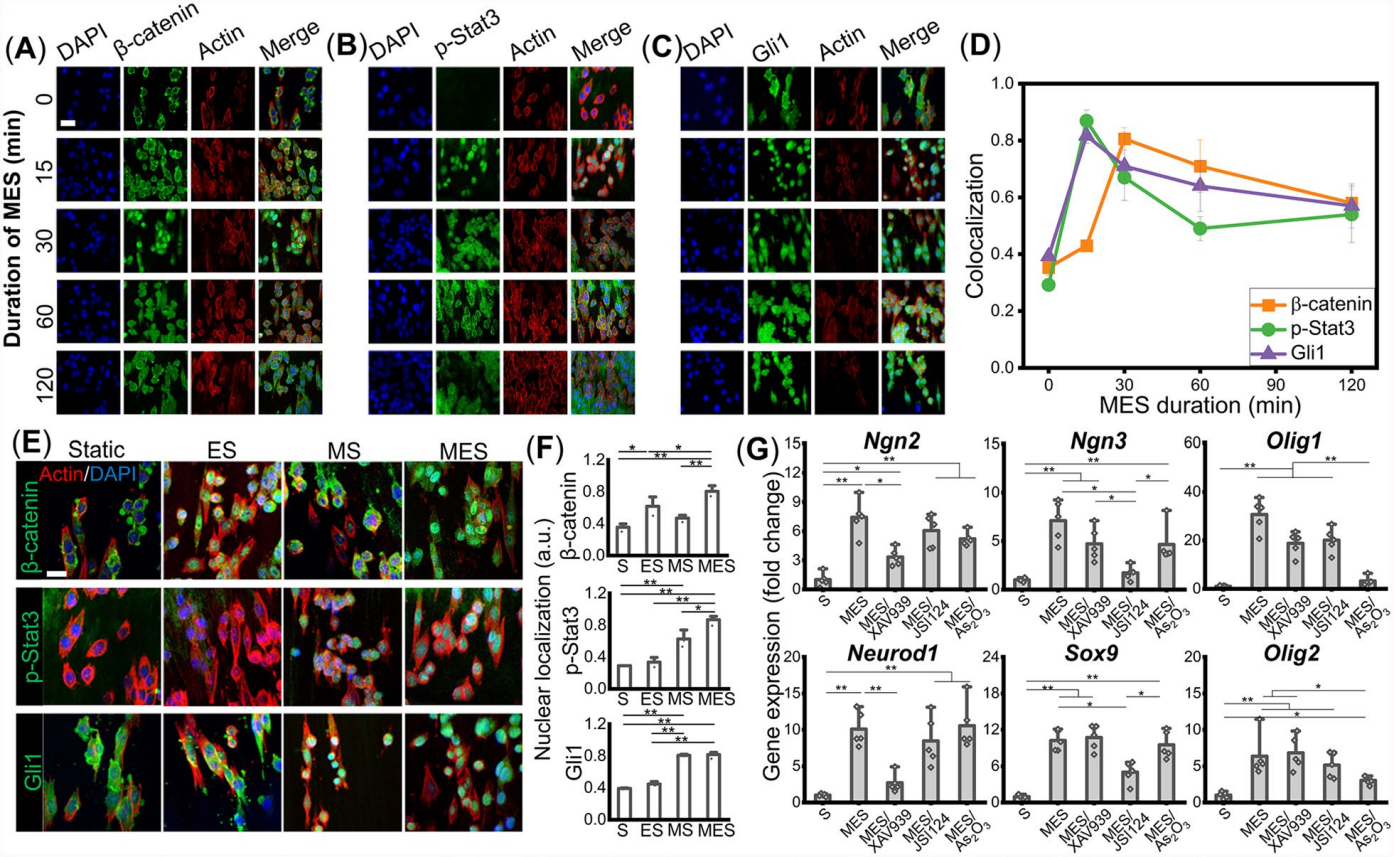
Wnt/β-catenin, JAK/Stat3, and Shh/Gli1 signaling pathways regulate mechano-electrical stimulation (MES)-induced neuronal, astrocytic, and oligodendrocytic differentiation of neural stem cells (NSCs), respectively. (**A**–**C**) Time course study of nuclear localization of (**A**) β-catenin, (**B**) p-Stat3, and (**C**) Gli1, the signature mediators of Wnt, JAK, and Shh signaling pathways, respectively, under MES (scale bar: 20 μm). (**D**) Quantification of MES duration-dependent nuclear localization of β-catenin, p-Stat3, and Gli1 (n = 3). (**E**) Immunofluorescence images of β-catenin, p-Stat3, and Gli1, in the Static (S), electrical stimulation (ES), mechanical stimulation (MS), or MES condition (scale bar: 25 μm) and (**F**) their quantification of nuclear localization (n = 3). (**G**) Gene expression levels of early neurogenesis markers *Ngn2*, *Neurod1*, early astrogenesis markers *Ngn3*, *Sox9*, and early oligodendrogenesis markers *Olig1*, *Olig2* after the application of MES in the presence of XAV939, JSI124 or As_2_O_3_, an inhibitor for Wnt, JAK, or Shh signaling, respectively. The Static and MES without any inhibitors were included as negative and positive controls, respectively (n = 5). * and ** denote statistical significance of *p* < 0.05 and *p* < 0.01, respectively

Previous studies have shown that the canonical Wnt/β-catenin, JAK/Stat3, and Shh/Gli1 signaling pathways significantly regulate the neurogenesis, astrogenesis, and oligodendrogenesis of NSCs, respectively, by controlling the transcription factors that we examined above [21–26, 31–33]. The nuclear localization of β-catenin, p-Stat3, and Gli1, which are the key mediators involved in the canonical Wnt, JAK, and Shh signaling pathways, respectively, were observed at various time durations within the 2 h of the MES to capture the transient signaling activation (Fig. 2A–C). Based on the results, we selected 30 min after the start of the MES for β-catenin, and 15 min for p-Stat3 and Gli1 nuclear localization for this study (Fig. 2D). We next examined the effects of ES, MS, and MES on these signaling pathways to investigate how they mediate MES-induced NSC multi-phenotypic differentiation. Interestingly, the nuclear translocation of β-catenin was observed in both ES and MES conditions while MS did not activate the Wnt signaling, as compared to the Static control, which showed the cytoplasmic localization of β-catenin (Fig. 2E, F). In contrast, the MS and MES conditions induced the activation and nuclear localization of the key transcription factor of the JAK signaling pathway, p-Stat3, while negligible expression of p-Stat3 was observed under the ES condition (Fig. 2E, F). Similarly, the nuclear localization of a Shh signaling transcription factor, Gli1, was observed under the MS and MES conditions (Fig. 2E, F). These results demonstrate that ES activates the Wnt/β-catenin signaling while MS initiates the JAK/Stat3 and Shh/Gli1 signaling pathways, collectively leading to the simultaneous differentiation of NSCs towards neurons and glial cells under MES.

To confirm the regulation of the phenotype-specific differentiation of NSCs via these signaling pathways under MES, an inhibitor for each Wnt/β-catenin, JAK/Stat3, or Shh/Gli1 signaling pathway was utilized. The gene expression levels of various differentiation markers were examined under MES in the absence or presence of the inhibitors. The optimal incubation duration of each signaling inhibitor was experimentally determined to efficiently inhibit the protein localization of β-catenin, p-Stat3, or Gli1 while not significantly affecting cellular morphology and viability (figure S2). The inhibition of Wnt/β-catenin signaling using XAV939, a Wnt antagonist, significantly reduced the expression levels of neurogenic transcription factors *Ngn2* and *Neurod1*, which were upregulated by MES (Fig. 2G). The MES-induced upregulation of astrocytic transcription factors *Ngn3* and *Sox9* were significantly downregulated by the inhibition of the JAK/Stat3 signaling through Cucurbitacin I (JSI124, Stat3 inhibitor). Similarly, significant downregulation of oligodendrocytic transcription factors that were upregulated under MES was observed only when the Shh signaling was inhibited by As_2_O_3_, a Gli antagonist (Fig. 2G). These inhibitors exhibited minimal effects on the multi-phenotypic differentiation of NSCs other than the specific phenotype regulated by the target signaling pathway. Collectively, these results demonstrate that neurogenesis, astrogenesis, and oligodendrogenesis under MES are mainly regulated by the signaling pathways of Wnt/β-catenin, JAK/Stat3, and Shh/Gli1, respectively, while no evident crosstalk effect among them was observed. A similar regulation of these signaling cascades under MES was observed in the multi-phenotypic differentiation of human NSCs (figure S3).

### Akt and ROCK signaling mediate electrical and mechanical stimulation-induced multi-phenotypic differentiation of neural stem cells.

**Fig. 3:**
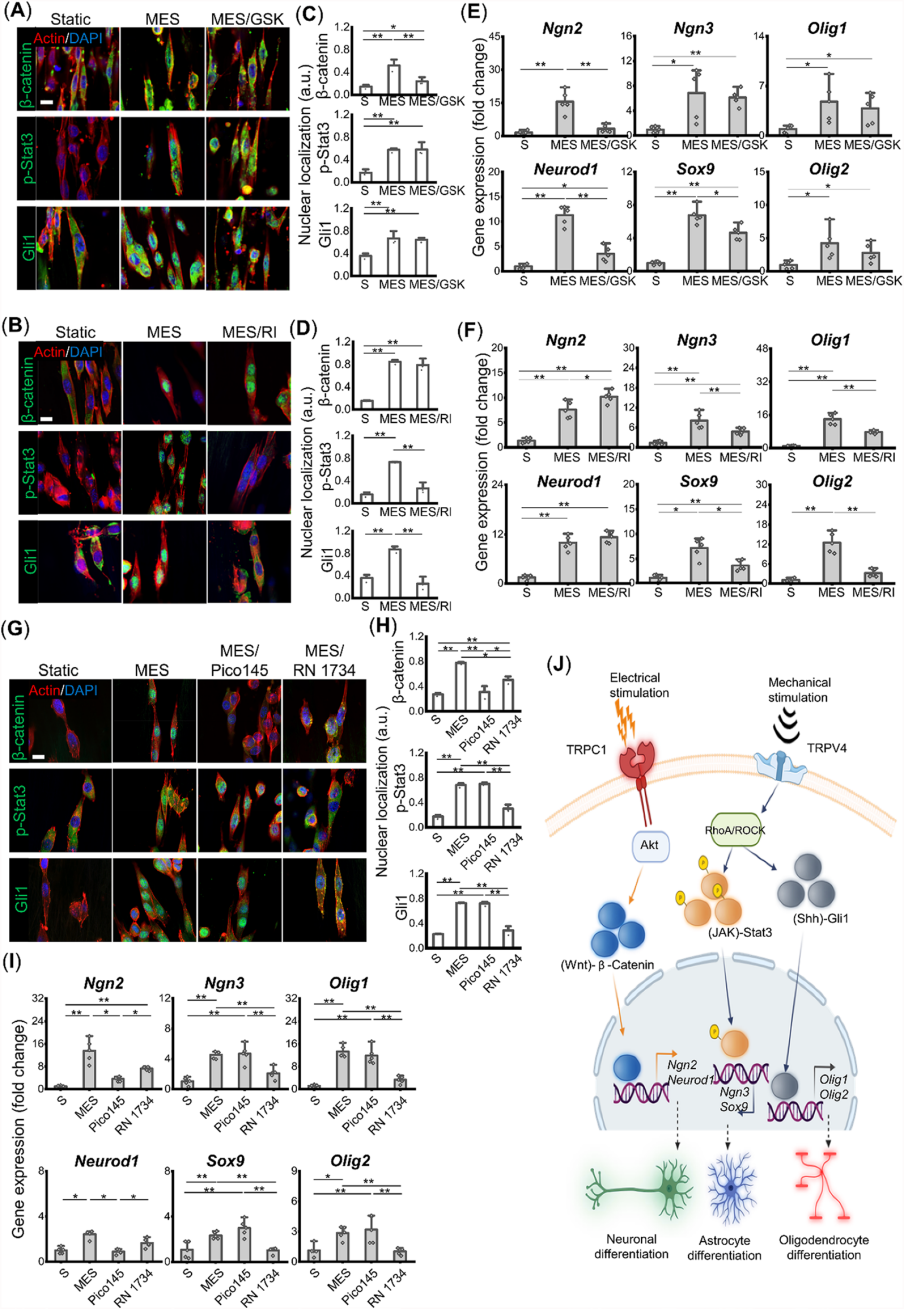
TRPC1-Akt and TRPV4-ROCK signaling axes mediate mechano-electrical stimulation (MES)-induced neuronal and glial differentiation of neural stem cells (NSCs). (**A**, **B**) Immunofluorescence images of β-catenin, p-Stat3, and Gli1 after the application of mechano-electrical stimulation in the absence or presence of the (**A**) Akt signaling inhibitor (GSK 690693, GSK), (**B**) ROCK signaling inhibitor (Y27632, RI). The Static (S) and MES conditions were used as negative and positive controls, respectively (scale bar: 10 μm). (**C**, **D**) Quantification of nuclear localization of the signature signaling mediators (n = 3). (**E**, **F**) Gene expression of early neurogenesis, astrogenesis, and oligodendrogenesis markers under the application of MES in the absence or presence of the various signaling inhibitors (n = 5). (**G**) Immunofluorescence images and (**H**) their quantification of β-catenin, p-Stat3, and Gli1 nuclear localization after the application of mechano-electrical stimulation in the absence or presence of the inhibitors for physical stimuli-responsive membrane channels including TRPC1 (Pico145) and TRPV4 (RN 1734). (**I**) Gene expression of early neurogenesis, astrogenesis, and oligodendrogenesis markers under the application of MES in the absence or presence of the various membrane channel inhibitors (n = 5). (**J**) A schematic depicting potential molecular mechanisms for the MES-induced multi-phenotypic differentiation of neural stem cells (NSCs). * and ** denote statistical significance of *p* < 0.05 and *p* < 0.01, respectively

The protein kinase B (Akt) has been shown to play important roles in regulating neuronal differentiation and is a key regulator of beta-catenin activity [34–36], while ROCK is a key mechano-mediator that participates in various cellular differentiation behaviors [37, 38]. We examined whether Akt and ROCK are required for the ES-induced neuronal differentiation and MS-induced glial differentiation of NSCs, respectively. The nuclear localizations of β-catenin, p-Stat3, and Gli1 were evaluated by immunofluorescence imaging and its quantification under various culture conditions including the Static, MES, and MES in the presence of an Akt inhibitor (GSK690693) or a ROCK inhibitor (Y27632) (Fig. 3A–D). The results showed a loss of β-catenin nuclear translocation by the inhibition of Akt under the MES condition and decreased p-Stat3/Gli1 nuclear translocation by the ROCK inhibition. Similarly, the expression of neuronal differentiation markers was significantly downregulated under MES in the presence of the Akt inhibitor while NSC differentiation toward other phenotypes was mostly not affected, indicating the involvement of Akt in the MES-induced neuronal differentiation (Fig. 3E). The inhibition of the mechano-signaling cascades, on the other hand, downregulated the expression of gliogenic genes (Fig. 3F), indicating the important role of ROCK for the MES-induced glial differentiation. We also found that there was little or no effect of ROCK inhibition on neuronal differentiation, demonstrating minimal signaling crosstalk between neurogenesis and gliogenesis.

### TRPC1 and TRPV4 channels are required for physical stimulation-induced neuronal and glial differentiation, respectively

The regulatory roles of TRP channels on multi-phenotypic differentiation of neural stem cells under MES were examined. TRPC channels and TRPV channels have been shown to be involved in various neural activities [39–42]. In addition, studies have shown that TRPA1, TRPM3, and TRPM7 channels exhibit mechanosensitivity [43]. Therefore, we performed a gene expression analysis using PCR electrophoresis to screen TRP channels that are expressed in neural stem cells. The expression of TRPC1-7, TRPV1-6, TRPA2, TRPM3, and TRPM7 channels was examined, and it was demonstrated that mNSCs are rich in the expression of TRPC1, TRPC2, TRPV4, and TRPM7 channels (figure S4A, B). Based on these results, inhibitors of TRPC1 (Pico145), TRPV4 (RN 1734), and TRPM7 (Ver 155,008) were used to determine which TRP channels were responsible for the MES-induced mNSC differentiation. It is noted that TRPC2 was excluded from analysis since it is encoded by a pseudogene in humans [44]. Immunofluorescence imaging results showed that the inhibition of TRPC1 induced a loss of MES-induced β-catenin nuclear translocation while the nuclear localization of p-Stat3 and Gli1 under MES were not significantly affected by TRPC1 inhibition (Fig. 3G, H). Interestingly, the inhibition of TRPV4 showed an opposite trend where the loss of p-Stat3 and Gli1 nuclear localization was observed while β-catenin expression was still maintained within the cell nuclear under MES (Fig. 3G, H). The inhibition of TRPM7, on the other hand, did not influence the expression pattern of the target proteins (figure S4C, D). Corroborating with these results, the TRPC1 channel played a major role in regulating neurogenic genes and TRPV4 mainly controlled the expression level of gliogenic genes, while there were no significant gene regulations through the TRPM7 channel (Fig. 3I, figure S4E).

Combining our results and evidence from previous studies, which showed that TRPC1 regulates Akt and TRPV4 directly activates RhoA [45–47], our mechanistic study uncovered signaling cascades involved in the MES-induced multi-phenotypic differentiation of NSCs (Fig. 3J); electrical stimulation induces neuronal differentiation of NSCs through sequential activation of TRPC1 channel, Akt, Wnt/β-catenin signaling pathway, and subsequent downstream neurogenic genes. On the other hand, mechanical stimulation activates mechanosensitive RhoA/ROCK signaling through the activation of the TRPV4 channel. The activation of ROCK further relays downstream signaling through JAK/Stat3 and Shh/Gli1, which induces astrocytic and oligodendrocytic differentiation, respectively.

### Selective regulation of physico-responsive signaling cascades enhances neuromorphogenesis

**Fig. 4:**
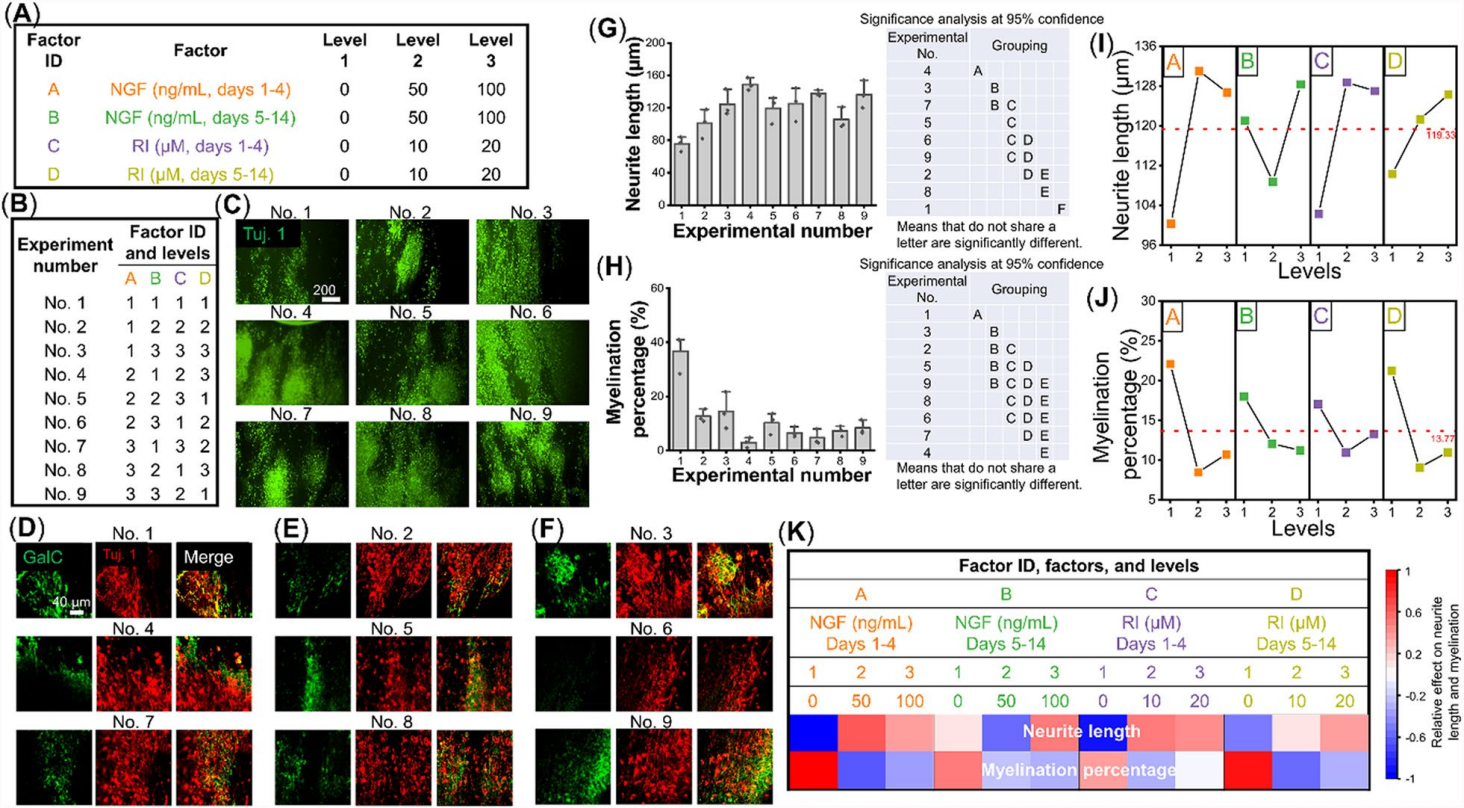
Taguchi robust parameter design (RPD) is utilized to determine parameters for the optimal application of biochemical factors to augment mechano-electrical stimulation (MES)-induced neuromorphogenesis. (**A**) Concentration and application duration of biochemical factors to control Akt and ROCK signaling pathways were investigated in this parameter optimization. 2 types of biochemical factors (Nerve growth factor (NGF) and ROCK inhibitor (RI)) with 2 different durations were assigned to 4 factor IDs while 3 concentrations under each factor ID were labeled as 3 levels. (**B**) L_9_ array with assigned factor IDs and levels. (**C**–**F**) Representative fluorescent images showing the (**C**) neurite and (**D**–**F**) myelination marker expression of each condition under the L9 array Taguchi design, where neurite length and myelination percentage were used as readouts for the parameter optimization. (**G**, **H**) Quantification and significance analysis of (**G**) neurite length and (**H**) myelination percentage of each condition under the L_9_ array Taguchi design (n = 3). (**I**, **J**) Results of RPD analysis for (**I**) neurite length and (**J**) myelination percentage to determine optimal conditions. (**K**) A heatmap describing the relative effect of each individual factor on neurite length and myelination

Based on the knowledge gained from the mechanistic study, several biochemical factors that regulate neuro- or gliogenesis were employed to further augment the MES-induced neuromorphogenesis of NSCs. Several studies have shown that neurite outgrowth, a key cellular behavior that would induce synaptic network formation and subsequent nerve tissue maturation [48, 49], is significantly inhibited by existing myelinating glia [50]. In order to develop an extensive neural network, it is, therefore, necessary to inhibit the glial cell development at an early stage of the MES-induced neuromorphogenesis while promoting neurite extension. In this regard, we utilized the RI to suppress the MES-induced activation of JAK/Stat3 and Shh/Gli1 signaling pathways, thus gliogenesis while a neurotropic nerve growth factor (NGF), which is a Wnt signaling agonist for neurite development [51, 52], was used to directly enhance the neurite outgrowth, in addition to the application of MES. To determine the optimal biochemical conditions, various concentrations and administration durations of NGF and RI were screened using the Taguchi RPD method. The experimental procedures are shown in figure S5, including the creation of an L_9_ orthogonal array with defined factors and levels, data collection and subsequent robust parameter analysis, and employment of the optimized parameters for the functional improvement of in vitro nerve tissue models. As shown in Fig. 4A, B, the concentration and duration of NGF and RI administration were designed as 2 factors and 3 levels to develop the L_9_ orthogonal array. From the result of the RPD analysis, based on the quantification of fluorescent images for neurites and myelination (Fig. 4C–H), the optimal parameters to achieve the maximum neurite length or the maximum myelination coverage could be selected, respectively (Fig. 4I, J). As expected, the optimal condition for maximum neurite length would result in the least myelination while the enhanced myelination would lead to poor neurite outgrowth (Fig. 4I, J). This is further demonstrated by examining the relative effect of factors and levels on the neurite length and the level of myelination, where they were inversely regulated under each factor (Fig. 4K).

**Fig. 5:**
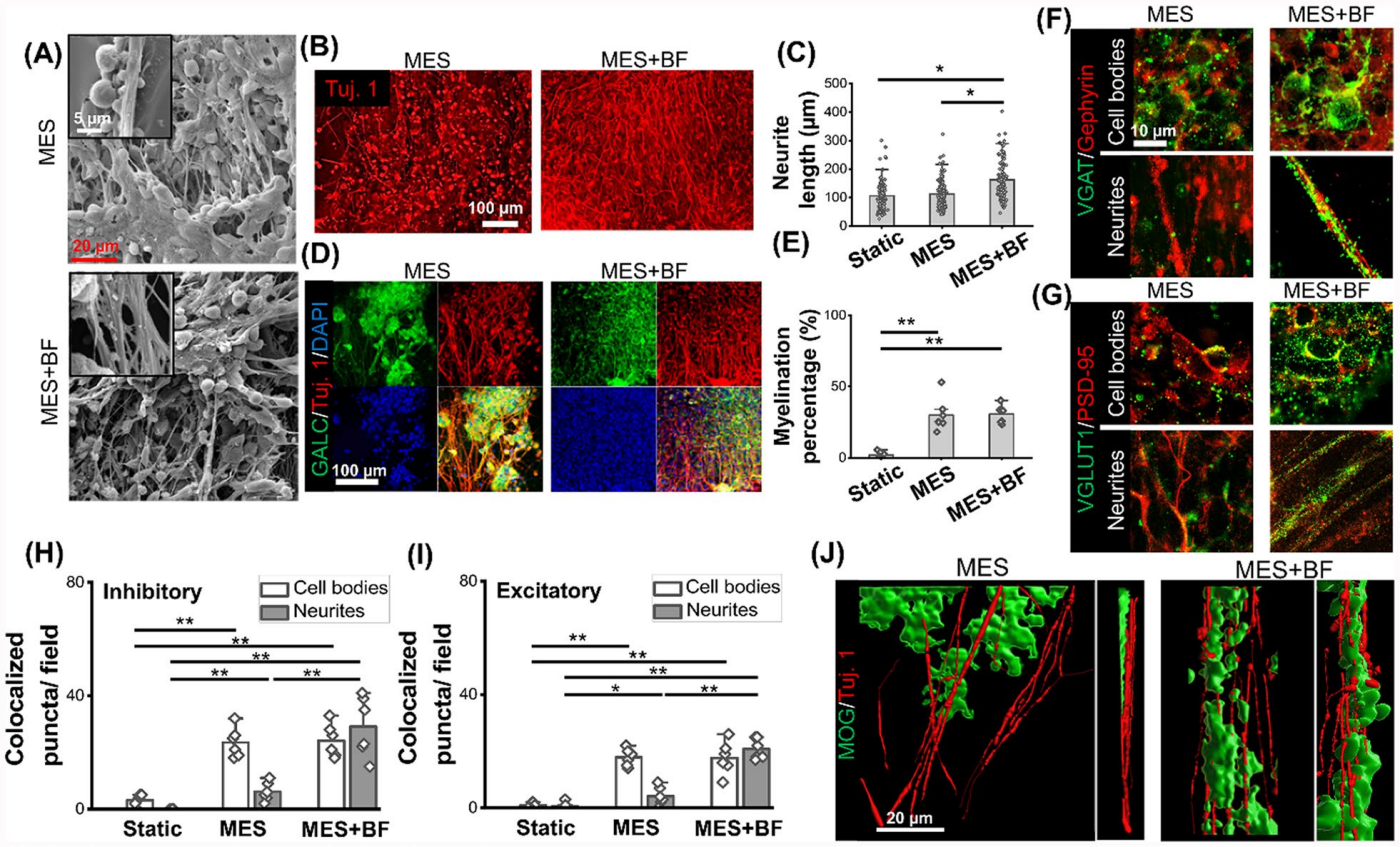
The application of biochemical factors augments the mechano-electrical stimulation (MES)-induced functional development of engineered nerve tissues from human neural stem cells (hNSCs). (**A**) Representative scanning electron microscopy (SEM) images showing the nerve tissue structure developed under the MES condition or the MES condition with biochemical factors (MES + BF). (**B**) Fluorescence images showing neurite network from the nerve tissues developed under the MES and MES + BF conditions. (**C**) Quantification of neurite length under various conditions including Static, MES, and MES + BF (n = 6, a total of 130 data points were presented in the graph from 6 independent samples under the same condition). (**D**) Confocal images showing the neurite and myelination structure under the MES and MES + BF conditions. (**E**) Quantification of the myelination percentage under various conditions including Static, MES, and MES + BF (n = 6). (**F**, **G**) Confocal images for (**F**) inhibitory (VGAT/Gephyrin) and (**G**) excitatory (VGLUT1/PSD95) synaptic markers. (**H**, **I**) Quantification of the number of colocalized pre- and post-synaptic puncta under various conditions (n = 6). (**J**) 3D reconstruction of confocal images immunofluorescently labeled by mature myelination marker MOG (green) and neuronal marker Tuj. 1 (red) under the MES and MES + BF conditions. * and ** denote statistical significance of *p* < 0.05 and *p* < 0.01, respectively. Images for the Static condition are presented in figure S7

Based on the results from the RPD analysis, A2, B3, C2, and D3 (50 ng/mL of NGF from day 1 to day 4, 100 ng/mL of NGF from day 5 to day 14, 10 µM of RI from day 1 to day 4, and 20 µM of RI from day 5 to day 14) were selected to achieve the development of long neurites. After the initial 14 days of optimal biochemical stimulation with MES, 3 additional weeks were used to apply only MES, without any biochemical factors. Such an extended culture duration was utilized to validate the formation of a mature neuronal network, induced by both MES and optimal biochemical stimulation, as well as to examine whether the extended culture period helps neurites regain myelination, which was lacking under the application of biochemical factors (BFs). More elongated projections, likely to be neuronal phenotypes, were observed after a 5-week culture period of MES without or with biochemical factors for the first 2 weeks (MES or MES + BF), as compared to the cells under the Static condition (Fig. 5A, figure S6A). Neuronal marker Tuj. 1 revealed significantly longer neurites under the MES + BF condition, as compared to the Static or MES condition (Fig. 5B, C, figure S6B). While there was a decrease in myelination after 2 weeks of biochemical factor application as shown in the RPD analysis (Fig. 4J), an extended 5-week culture duration showed no difference in the degree of myelination between the MES and the MES + BF conditions (Fig. 5D, E). Corroborating with Fig. 1A–C, however, there was no sign of glial cells even after 5 weeks of culture under the Static condition (figure S6C). We further examined synaptic markers and mature myelination markers to determine the functional maturity of the engineered tissues under different culture conditions. A similar synaptic connection, indicated by the number of colocalized puncta of VGAT and post-inhibitory synaptic marker Gephyrin, was observed around cell bodies between the MES and MES + BF conditions. On neurites, however, there were significantly more inhibitory synaptic interactions observed under the MES + BF condition (Fig. 5F, H). Similar to the inhibitory synapses, excitatory synaptic markers (pre-synaptic marker VGLUT1 and post-synaptic marker PSD-95) were significantly more co-localized under the MES + BF condition on neurites, while no significant difference was observed around cell bodies (Fig. 5G, I). Note that no interactions of synapses were observed under the Static condition (figure S6D). Interestingly, unlike the early myelination marker GalC, there was a different expression pattern of the mature myelination marker, MOG, between the MES and MES + BF conditions. As shown in 3D Imaris reconstruction images from Z-stack confocal imaging, the MES condition exhibited a layered cellular structure of MOG^+^ and Tuj. 1^+^ cells while the MES + BF condition resulted in a mixed structure of these two cell phenotypes, likely indicating a closer interaction between neurons and mature oligodendrocytes under the MES + BF condition (Fig. 5J, figure S6E, figure S7A-C).

## Discussion

While there have been extensive attempts and applications of utilizing various forms of physical stimuli, including electrical and mechanical stimulation, to regulate the differentiation behaviors of stem cells, the underlying signaling mechanisms that mediate such physical stimulation-induced cellular behaviors have not been systematically understood. Such limited mechanistic understanding often caused delamination between physical-driven- and biochemical-driven-nerve tissue model development even though both the physical environment and biochemical cues are prerequisites for the maturation and functional development of nerve tissues in vivo. In this regard, our study utilized a piezoelectric cell culture system with optimal electrical and mechanical stimulation regimens to successfully induce the multi-phenotypic differentiation of NSCs, followed by a systematic approach to unravel the mechanistic details of the mechano-electrical stimulation-induced stem cell differentiation. Furthermore, based on the knowledge gained from the mechanistic study, several molecules mediated in the physical stimulus-responsive signaling cascades were biochemically targeted to further enhance the functional maturation of engineered nerve tissues under MES.

The effects of MES on the multi-phenotypic differentiation of NSCs were decoupled into a single stimulus of ES or MS to reveal relatively independent roles of electrical and mechanical stimulation on NSC differentiation; ES induces neuronal differentiation while MS promotes glial differentiation. Based on these observations, a hypothesis was established that ES and MS activate independent signaling cascades to induce NSC differentiation toward specific phenotypes. Previous studies revealed that Wnt/β-catenin signaling controls neuronal differentiation in NSCs both in vivo and in vitro [27] while JAK/Stat3 and Shh/Gli1 signaling cascades are critical mediators for astrocytic and oligodendrocytic differentiation, respectively [21, 22, 24, 25]. In this study, we revealed the electrical and mechanical sensitivity of Wnt/β-catenin, JAK/Stat3, and Shh/Gli, where the application of ES activates β-catenin while the application of MS induces Stat3 and Gli1 activation in neural stem cells. Thus, the simultaneous activation of β-catenin, Stat3, and Gli1 under MES results in the multi-phenotypic differentiation of NSCs towards neurons, astrocytes, and oligodendrocytes, respectively. These results collectively demonstrate the potency of MES in activating multiple signaling cascades and inducing simultaneous neuronal and glial differentiation of NSCs.

Several studies showed that electromagnetic fields can activate the TRPC channels in NSCs, inducing their neuronal differentiation while the mechano-responsive TRPV channels are involved in neural cell differentiation and proliferation behaviors [39, 53, 54]. However, mechanistic details about how these receptors under physical stimuli subsequently regulate neuro- and gliogenesis are currently unknown, leading us to investigate mechanisms linking physical stimuli-responsive membrane channels to subsequent intracellular signaling cascades. After confirming the strong expression of the TRPC1 channel and the TRPV4 channel in mNSCs, we then validated that the TRPC1 channel is electrically responsive and required for stimulation-induced neuronal differentiation. More interestingly, we found that β-catenin, regulated by Akt, was a key component in mediating the ES-TRPC1-neuronal differentiation cascade. On the other hand, the TRPV4 channel, which acts upstream of both JAK/Stat3 and Shh/Gli1 signaling, is required for MS-induced glial differentiation, mediated by the mechano-transduction RhoA/ROCK signaling pathway. These observed signaling cascades corroborate with previous studies on other cell types. For example, TRPC activates the Akt molecule, resulting in the activation of β-catenin in neuroblastoma cells [55]. Similarly, RhoA/ROCK is controlled by the TRPV4 channel and the activation of RhoA/ROCK has been shown to regulate downstream signaling transcription factors including Stat3 and Gli1 in amoeboid cancer cells and basal cell carcinomas, respectively [45, 46, 56–58]. Our study was the first to establish a systematic signaling network describing the mechanism of physical stimuli-induced neuronal and glial differentiation of NSCs, conserved across different species as confirmed in both human and mouse NSCs. It should be noted that potential synergistic effects of ES and MS were observed, which requires careful examination in the future.

Although MES induces simultaneous differentiation of NSCs towards neurons and glial cells, the limited neurite outgrowth potentially leads to the development of unnaturally short fragments in the neural circuit, resulting in insufficient synaptic network formation [48, 49]. Therefore, based on the identified signaling network, we incorporated two biochemicals, NGF and ROCK inhibitor into the MES stem cell culture regimen, to activate/deactivate specific signaling cascades for enhanced functional maturation of engineered nerve tissues. NGF has been shown to enhance neurite outgrowth by inhibiting GSK-3 activity [52]. The inhibition of GSK-3 activity reduces the phosphorylation of β-catenin, leading to its enhanced nuclear translocation [59]. This mediation of NGF-induced neuronal development through β-catenin corroborates our finding that electrical stimulation enhances neurogenesis through Wnt/β-catenin signaling. Therefore, NGF was employed to further enhance neuronal cell development. In addition, it was reported that the presence of glial cells inhibits neurite outgrowth and neuronal maturation at the nerve injury site where glial scar tissue typically forms [60, 61]. Hence, the application of ROCK inhibitor was utilized to inhibit the MES-induced glial differentiation to potentially result in extended neurite outgrowth.

To systematically optimize the concentration and duration of the two biochemicals, the Taguchi method of RPD was employed for efficient parameter optimization [62, 63]. A full factorial experiment for a 4-factor 3-level Design of Experiment (DOE) analysis requires a total number of 81 conditions while an L_9_ orthogonal RPD analysis only needs 9 conditions with reliable outcomes [64]. The results of the RPD analysis showed that the neurite outgrowth and myelination are inversely regulated by the combination of NGF and ROCK inhibitor, where cells with decent neurite outgrowth exhibited poor myelination and vice versa. For a more extensive neural network formation, we selected the condition that induced the longest neurite length for the stimulation regimen. An extended cell culture duration (first 2 weeks with biochemical supplementation with the application of MES, followed by an additional 3 weeks under MES) was also utilized to help regain myelination. As expected, a significant increase in neurite outgrowth, along with more enriched inhibitory and excitatory synaptic connections, was observed after incorporating the biochemicals into the MES-induced NSC differentiation scheme. More significantly, we noticed a much closer interaction between mature myelinating oligodendrocytes and neurons when the biochemicals were utilized to augment neuromorphogenesis; intermingled cell populations of neurons and oligodendrocytes were observed under the incorporation of biochemicals while MES alone resulted in a layered structure of neurons and oligodendrocytes having a limited interface between those two cell types. This could be attributed to the fact that the early formation of an enriched neuronal network under the MES + BF condition further enhanced glial differentiation and mature myelination during the extended culture duration, even if the glial differentiation was largely inhibited at the early period due to the ROCK inhibitor. This is consistent with previous findings, which showed that differences in neuronal networks and neuronal activities typically result in different myelination behaviors from oligodendrocytes [65, 66].

## Conclusion

This study uncovered the signaling cascades that mediate the MES-induced multi-phenotypic differentiation of NSC towards neurons and glial cells. The results demonstrate that electrical stimulation induces neuronal differentiation of NSCs through sequential activation of the TRPC1 channel, Akt, and Wnt/β-catenin signaling axis to control downstream pro-neuronal genes. On the other hand, mechanical stimulation activates the mechanosensitive RhoA/ROCK pathway via the TRPV4 channel, resulting in the activation of downstream signaling pathways JAK/Stat3 and Shh/Gli1, ultimately regulating astrocytic and oligodendrocytic differentiation, respectively. The knowledge of such signaling mechanisms was further applied to the MES-induced differentiation scheme to enhance the functional maturation of engineered nerve tissues with extended neurite outgrowth, enriched synaptic interaction, and mature myelin formation. The functional enhancement of the nerve tissue model provides a means for broad future applications, such as physiologically relevant nerve injury model development, effective drug screening and mechanistic studies, and potential therapeutics for nerve tissues.

## Supplementary Information

Additional file 1

## Data Availability

The datasets used and/or analysed during the current study are available from the corresponding author on reasonable request.

## Abbreviations

NSC: Neural stem cell
CNS: Central nervous system
MES: Mechano-electrical stimulation
JAK: Janus kinase
Shh: Sonic hedgehog
TRP: Transient receptor potential channel
P(VDF-TrFE): Poly(vinylidene fluoride-trifluoroethylene)
PVDF: Polyvinylidene fluoride
iPSCs: Human induced pluripotent stem cells
PFA: Paraformaldehyde
RPD: Robust parameter design
RI: Rock inhibitor
SEM: Scanning electron microscopy
NGF: Nerve growth factor
ES: Electrical stimulation
MS: Mechanical stimulation
BF: Biochemical factor
