# Supplementary material for: Enhanced neuromorphogenesis of neural stem cells via the optimization of physical stimulus-responsive signaling pathways
Source: Stem Cell Res Ther. 2025 Jul 18;16:385. doi: 10.1186/s13287-025-04488-y (PMC12275341; doi:10.1186/s13287-025-04488-y)
Supplement: Supplementary file 1 — Additional file 1 [file 13287_2025_4488_MOESM1_ESM.pdf]

# Additional file

**Enhanced Neuromorphogenesis of Neural Stem Cells via the Optimization of Physical Stimulus-Responsive Signaling Pathways**

Youyi Tai[1], Natasha Brinkley[1], Lu Jin[1], Yu Wei Chang[1], Allen Liakhovetski[1], and Jin Nam[1]*

[1]Department of Bioengineering, University of California, Riverside, California, United States.

E-mail: jnam@engr.ucr.edu

**Table S1.** Mouse primers for the qRT-PCR of neurogenesis, astrogenesis, and oligodendrogenesis.

| Marker | Primer | Forward | Reverse |
|---|---|---|---|
| Housekeeping | *Gapdh* | 5'-GGGTGTGAACCACGAGAAAT-3' | 5'-TTCAGCTCTGGGATGACCTT-3' |
| Neuron | *Tubb3* | 5'-ACCTTGTGTCTGCCACCATGA-3' | 5'-CACCATGTTCACAGCCAGCTT-3' |
| | *Map2* | 5'-AAGCCATTGTGTCCGAACCA-3' | 5'-GAGCGGAAGAGCAGTTTGTCA-3' |
| | *Eno2* | 5'-ATCGCCACATTGCTCAGCTAG-3' | 5'-TGAGAGCCACCATTGATCACA-3' |
| | *Ngn1* | 5'-AAGCCATTGTGTCCGAACCA-3' | 5'-GAGCGGAAGAGCAGTTTGTCA-3' |
| | *Ngn2* | 5'-GTGCAGCGCATCAAGAAGA-3' | 5'-CGGCGTTTAGGTTGTGCAT-3' |
| | *Neurod1* | 5'-AAAGCCCCCTAACTGACTGCA-3' | 5'-TCAAACTCGGCGGATGGTT-3' |
| Astrocyte | *Aldh1l1* | 5'-AGTGATGTTGACAAGGCGGTG-3' | 5'-CGGTCACGCGCATTTATCTT-3' |
| | *Cspg5* | 5'-GTTGGCTTCGTCAGGCACAATG-3' | 5'-CCTTGTGCCAGATGTAGTCCTG-3' |
| | *Gfap* | 5'-TGGAGCTCAATGACCGCTTT-3' | 5'-GCTCGAAGCTGGTTCAGTTCA-3' |
| | *Ngn3* | 5'-TGGCATCATCGTCACAACGT-3' | 5'-CCCAGTTCGTCCATTTTTCG-3' |
| | *Nfia* | 5'-GCCGTGAAGGATGAACTGCTA-3' | 5'-TCTTTCCGTAACTTGGCCAGA-3' |
| | *Sox9* | 5'-AAGTCGGTGAAGAACGGACAA-3' | 5'-CAGCGCCTTGAAGATAGCATT-3' |
| Oligodendrocyte | *Nkx2.2* | 5'-CTGAGCAATCTGTCTTTCCCAGT-3' | 5'-TGTGTATGCAGAGGTTCAAGCCT-3' |
| | *Cldn11* | 5'-TGGCATCATCGTCACAACGT-3' | 5'-CCCAGTTCGTCCATTTTTCG-3' |
| | *Mog* | 5'-TGCCCTGCTGGAAGATAACAC-3' | 5'-TGCAGCCAGTTGTAGCAGATG-3' |
| | *Olig1* | 5'-ACGCCAAAGAGGAACAGCA-3' | 5'-TCCATGGCCAAGTTCAGGT-3' |
| | *Olig2* | 5'-GGCTTCAAGTCATCTTCCTCCA-3' | 5'-GGCTCAGTCATCTGCTTCTTGTC-3' |

**Table S2.** Human primers for the qRT-PCR of neurogenesis, astrogenesis, and oligodendrogenesis.

| Marker | Primer | Forward | Reverse |
|---|---|---|---|
| Housekeeping | *GAPDH* | 5'-ATGGGGAAGGTGAAGGTCG-3' | 5'-TAAAAGCAGCCCTGGTGACC-3' |
| Neuron | *TUBB3* | 5'-ACCTGGTATCGGCCACCATGA-3' | 5'-CACCATGTTGACGGCCAGCTT-3' |
| | *MAP2* | 5'-CTCCTCTGGCTTCCGATATTCTAAC-3' | 5'-AGGTGTGGTGGCTGGAAGGTAA-3' |
| | *ENO2* | 5'-ATCGCCACATTGCTCAGCTGG-3' | 5'-TGAGAGCCACCATTGATCACG-3' |
| | *NGN2* | 5'-CATCAAGAAGACCCGTAGACTGA-3' | 5'-TCTCGATCTTGGTGAGCTTGG-3' |
| | *NEUROD1* | 5'-AAAGCCCTCTGACTGATTGCA-3' | 5'-GGACGGTTCGTGTTTGAAAGA-3' |
| Astrocyte | *ALDH1L1* | 5'-CAAGTACTCCCGGTGGCGTG-3' | 5'-TGCAGAAGGGCAGGACGTTG-3' |
| | *CSPG5* | 5'-GCCCAGCTTCTGAACTCCCC-3' | 5'-CTTGAGGGTCGGGTGTGCTG-3' |
| | *GFAP* | 5'-CCAGTGAGCGGGCAGAGATG-3' | 5'-TGGTTCAGCTCAGCAGCCAG-3' |
| | *NGN3* | 5'-AAGCTCACCAAGATCGAGACG-3' | 5'-TACAAGCTGTGGTCCGCTATG-3' |
| | *SOX9* | 5'-GACTTCCGCGACGTGGAC-3' | 5'-GTTGGGCGGCAGGTACTG-3' |
| Oligodendrocyte | *NKX2.2* | 5'-ACGCAGGTCAAGATCTGGTTC-3' | 5'-GCGTCACCTCCATACCTTTCTC-3' |
| | *CLDN11* | 5'-GCTGGATCGGGGTCATCGTG-3' | 5'-GGATGGTGTAGCCGCAGGTC-3' |
| | *MOG* | 5'-CTCTGGTCGGGGATGAAGTG-3' | 5'-TCTCCATCTTGGTCCTTGCC-3' |
| | *OLIG1* | 5'-GCGAGGTCATCCTGCCCTACT-3' | 5'-CAGCAGTAGGATGTAGTTGCGG-3' |
| | *OLIG2* | 5'-GACAAGCTAGGAGGCAGTGG-3' | 5'-TCCGGCTCTGTCATTTGCTT-3' |

**Table S3.** Mouse primers for TRP channels.

| Primer | Forward | Reverse |
|---|---|---|
| *TRPC1* | 5'-CTCAGACATTCCAGGTTTCGTC-3' | 5'-CTCGTTTGTCAAGAGGCTCATC-3' |
| *TRPC2* | 5'-AGAGAGTGCAGAGCCCAGAG-3' | 5'-GAAGACAAGGAGGCAGGAGA-3' |
| *TRPC3* | 5'-CCTTGGGTCTTCCATTCCTC-3' | 5'-CACAACTGCACGATGTACTCC-3' |
| *TRPC4* | 5'-GTTGGGAGCTGCAAGAACTC-3' | 5'-TGGGCTGAGCAACAAACTC-3' |
| *TRPC5* | 5'-GATCATCCTCAACCATCGAGA-3' | 5'-GGAGAAGCATGAAGAGGAAGG-3' |
| *TRPC6* | 5'-TTAGCAATGAGCTGGCAGTG-3' | 5'-GCTGTTGCTGACAGTTTGGA-3' |
| *TRPC7* | 5'-TGTGCCGAGACACAGAAGAG-3' | 5'-ACTCCAAAGACAGCCAGGAA-3' |
| *TRPV1* | 5'-AGGGAGATCCACGAACCAG-3' | 5'-TAGTAAGCAGCCGTGGTGAA-3' |
| *TRPV2* | 5'-GTGGGATGTGGTGACCTACC-3' | 5'-GCTGGTACAGCCTGAGAAC-3' |
| *TRPV3* | 5'-TGGCCAAGGAAGAACAGAGGCAGAA-3' | 5'-TTCAGCGCTGTCTGCCCTTCAT-3' |
| *TRPV4* | 5'-TCTGTGCTGGTGGTTGTCTC-3' | 5'-GCTCAGCATCTCCAGGTCTC-3' |
| *TRPV5* | 5'-ACATGGTCTGCTTCACCACA-3' | 5'-GTAGCGAGAGGCACCAACTC-3' |
| *TRPV6* | 5'-TGAGCCTCAAGTGGAAGAGG-3' | 5'-CCCAACCTGAAGATGTCTGG-3' |
| *TRPA1* | 5'-GTTCAAAGCGGAGACTTGGA-3' | 5'-TAACGAGGCTCTGTGAAGCA-3' |
| *TRPM3* | 5'-GGAAATACGGAGCAGAGGTG-3' | 5'-AACAGCTGGTCCCTCAAAGA-3' |
| *TRPM7* | 5'-TACATGGAGGCATGCAGAAG-3' | 5'-CACCAAGATGAAATGGGAGTG-3' |

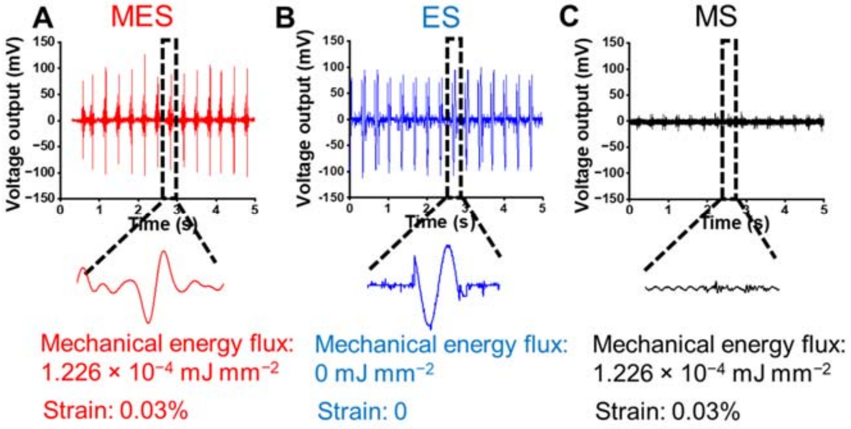


**Figure S1**. Representative electrical signal output generated from (A) piezoelectric scaffold (MES), (B) function generator (ES), or (C) piezoelectric-inactivated scaffold (MS).

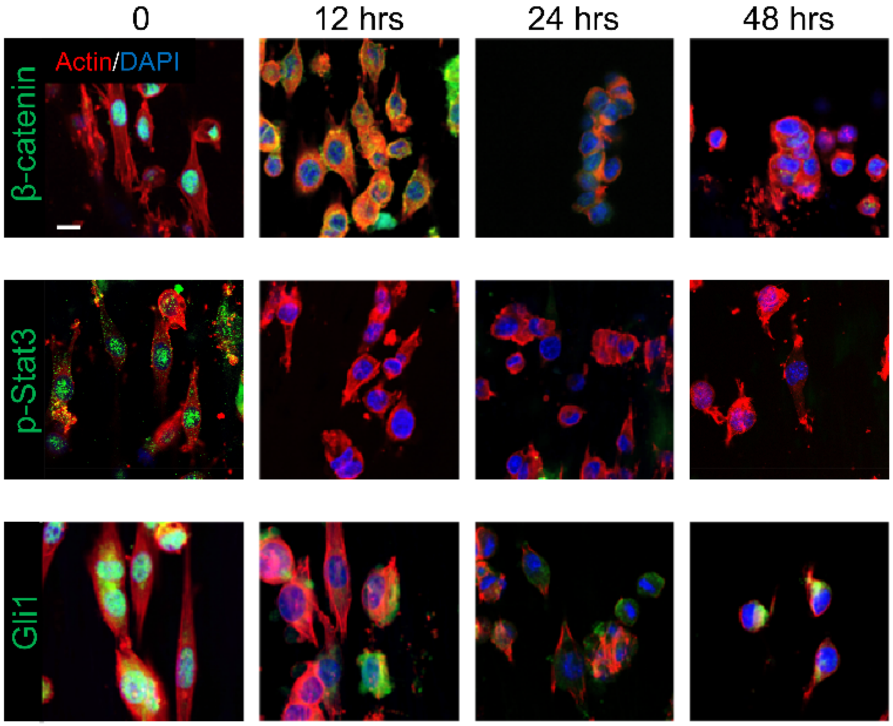


**Figure S2.** Incubating duration optimization of inhibitors for (top row) Wnt/β-catenin (XAV939), (middle row) JAK/Stat (Cucurbitacin I), and (bottom row) Shh ($As_2O_3$) signaling pathways.

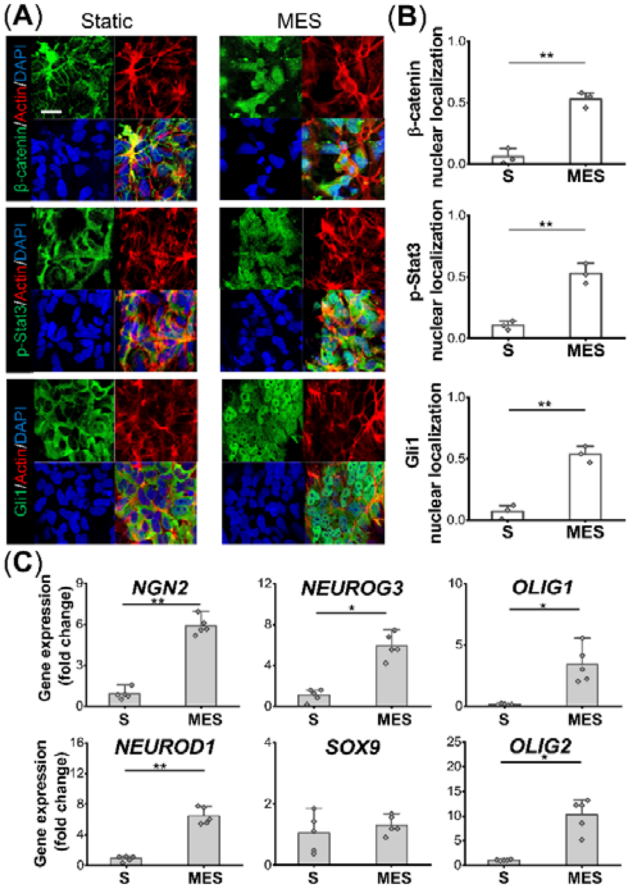


**Figure S3. Wnt, JAK, and Shh signaling mediate the mechano-electrical stimulation (MES)-induced multi-phenotypic differentiation of human neural stem cells (hNSCs).** (A) Immunofluorescence images of a Wnt signaling mediator β-catenin, JAK signaling mediator p-Stat3, and Shh signaling mediator Gli1 under the Static culture conditions or under the MES (scale bar: 10 μm). (B) Quantification of nuclear localization of the signature transcription factors (n=3). (C) Gene expression of early neurogenesis markers *Ngn2*, *Neurod1*, early astrogenesis markers *Ngn3*, *Sox9*, and early oligodendrogenesis markers *Olig1*, *Olig2* in statically cultured hNSCs or after 2 hours of MES, determined by RT-qPCR using the primers shown in **Table S2** (n=5). * and ** denote statistical significance of $p < 0.05$ and $p < 0.01$, respectively.

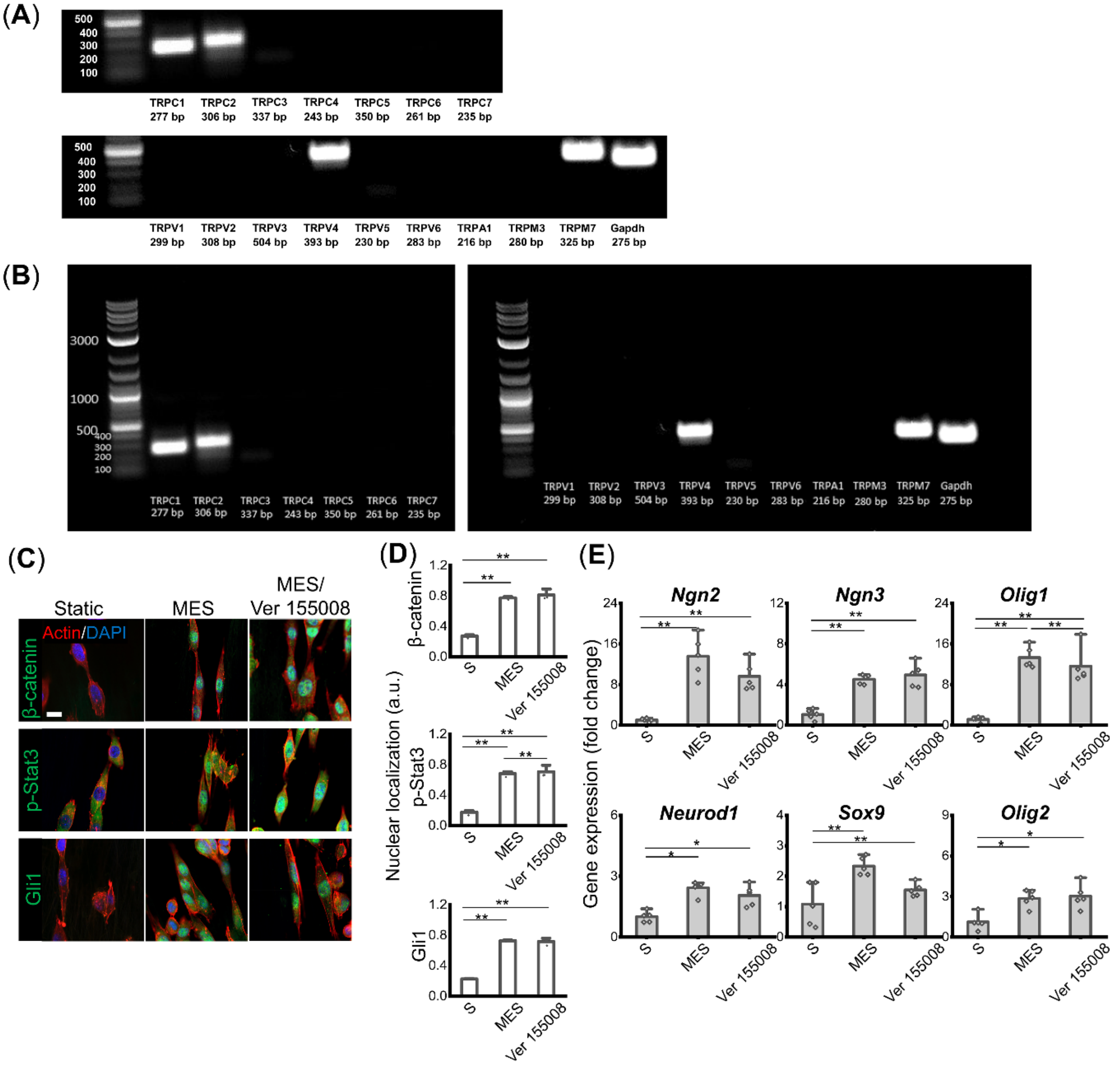


**Figure S4.** (A, B) A cropped, representative gel electrophoresis images illustrating mRNA expression of *TRPC1-7*, *TRPV1-6*, *TRPA1*, *TRPM3*, *TRPM7*, and *Gapdh* in mouse neural stem cells (mNSCs). Full-length blots/gels are presented in (B). (C) Immunofluorescence images and (D) their qunatification of β-catenin, p-Stat3, and Gli1 nuclear localization after the application of mechano-electrical stimulation in the absence or presence of the inhibitors for physical stimuli-responsive membrane channel TRPM7 (Ver 155008). (E) Gene expression of early neurogenesis, astrogenesis, and oligodendrogenesis markers under the application of MES in the absence or presence of the TRPM7 inhibitor (n=5).

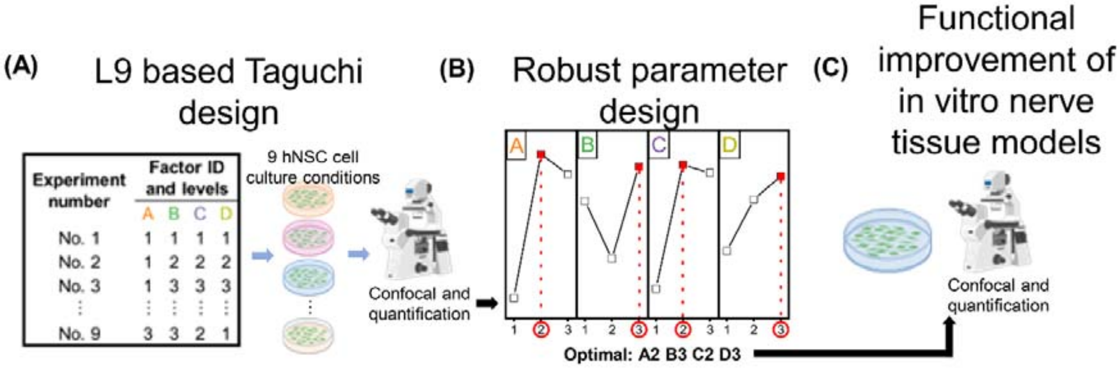


**Figure S5.** A schematic of the Taguchi process including (A) experimental array determination, (B) robust parameter design analysis, and (C) employment of the optimized parameters.

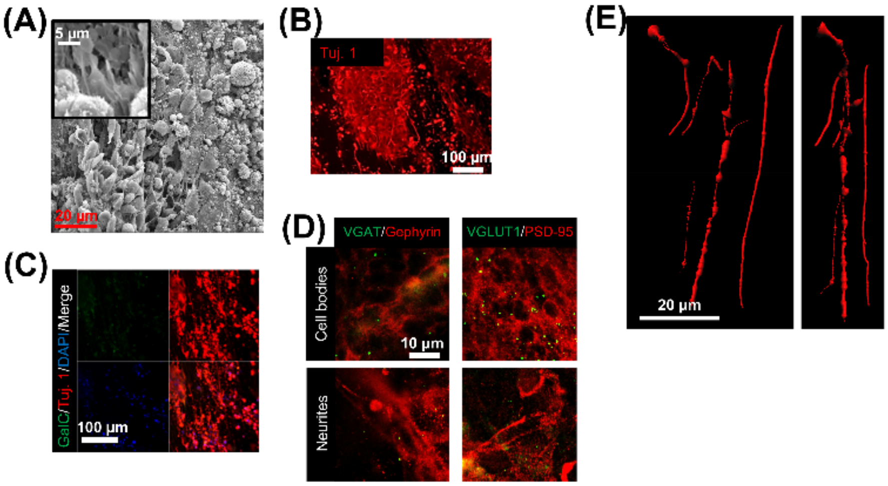


**Figure S6. Results of the Static condition where human neural stem cells (hNSCs) were cultured statically on electrospun aligned P(VDF-TrFE) scaffold for 5 weeks as a comparison with the mechano-electrical stimulation (MES) and the (MES with signaling factors) MES+BF conditions (Figure 5).** (A) Representative SEM image showing the cellular structure under the Static condition. (B) A fluorescence image showing neuronal differentiation from the Static condition. (C) Confocal image showing the detailed nerve and myelination structure under the Static condition. (D) Confocal images for examining inhibitory and excitatory neuronal synaptic markers under the Static condition. (E) 3D reconstruction of a confocal image fluorescently labeled by mature myelination marker MOG and neuronal marker Tuj. 1 under the Static condition.

(A)

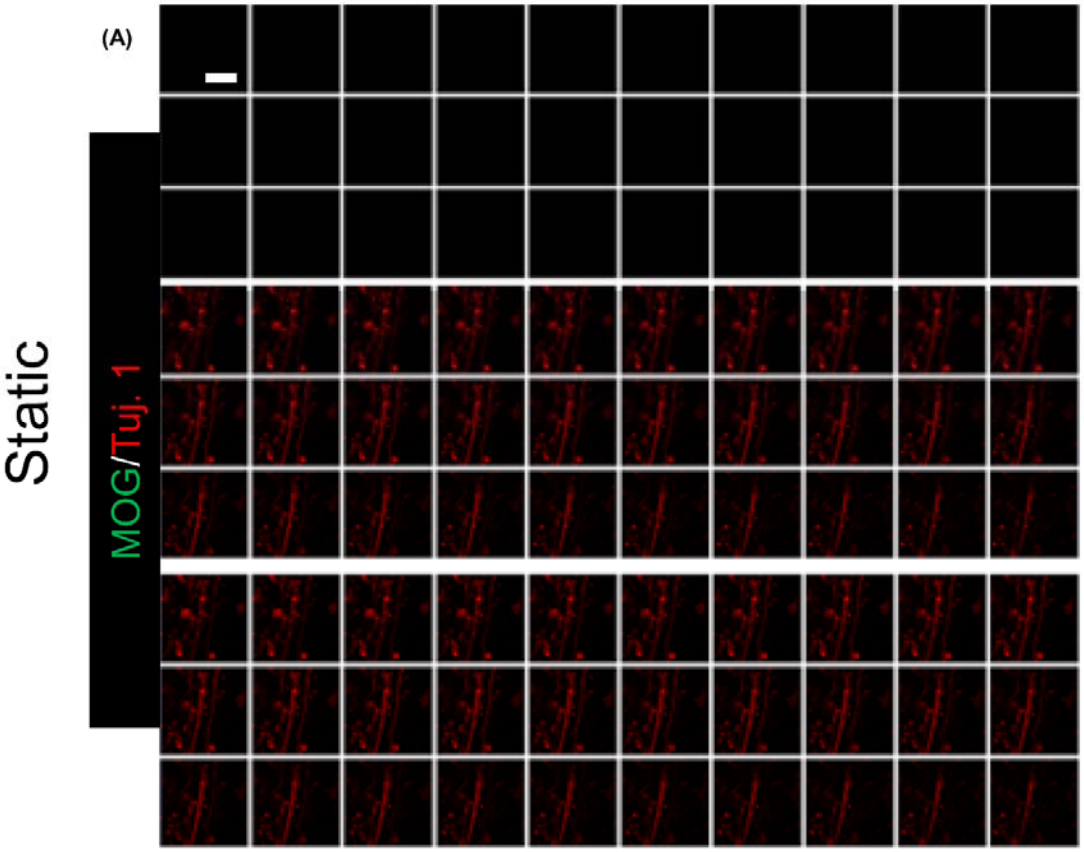

(B)

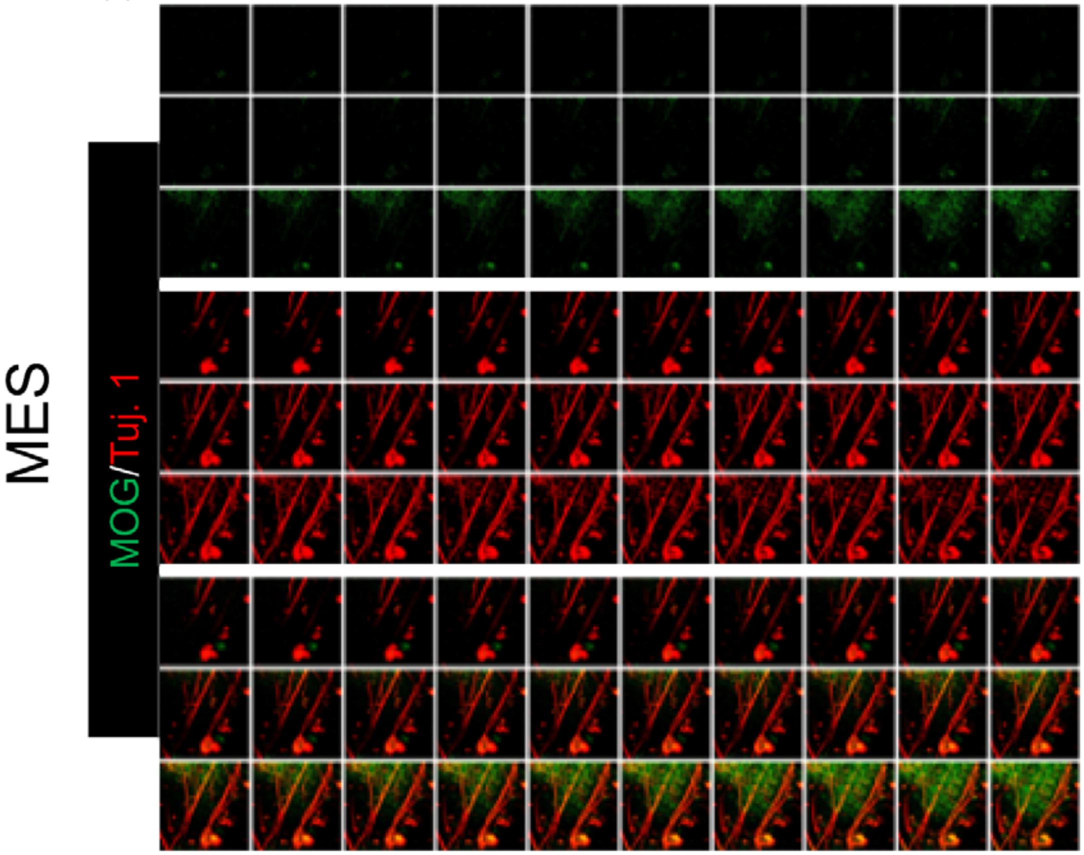

(C)

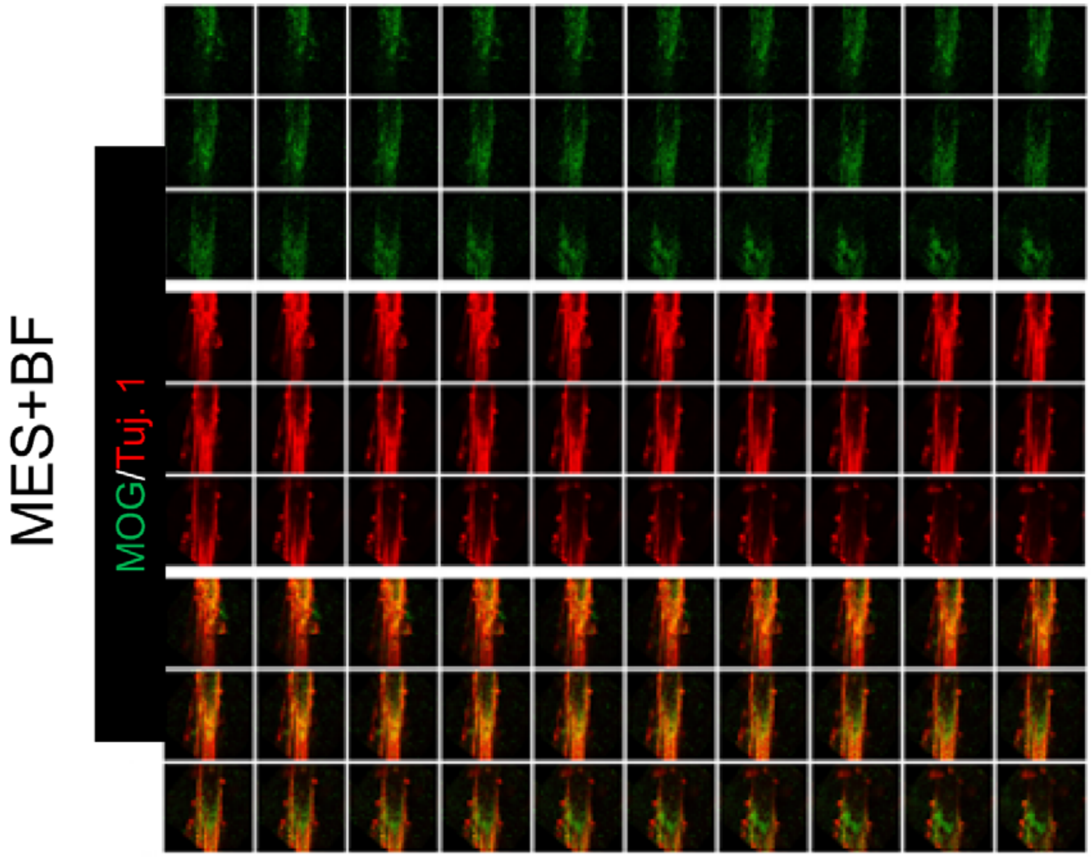


**Figure S7.** Z-stack confocal images showing the expression of mature myelination marker MOG and neuronal marker Tuj. 1 under the (A) Static, (B) mechano-electrical stimulation (MES), and (C) MES with signaling factors (MES+BF) conditions. Scale bar: 20 µm.
